# Elaboration and Characterization of Vitreous Fertilizers and Study of Their Impact on the Growth, Photosynthesis, and Yield of Wheat (*Triticum durum* L.)

**DOI:** 10.3390/ma14051295

**Published:** 2021-03-08

**Authors:** Tariq Labbilta, Mohamed Ait-El-Mokhtar, Younes Abouliatim, Mehdi Khouloud, Abdelilah Meddich, Mohamed Mesnaoui

**Affiliations:** 1 Laboratory of Materials Sciences and Processes Optimization, Chemistry of Condensed Matter and Environment Team, Chemistry Department, Faculty of Sciences Semlalia, Cadi Ayyad University, Marrakech 40000, Morocco; mesnaoui@uca.ac.ma; 2 Laboratory of Agro-Foods, Biotechnologies and Valorisation of Bioressources Vegetales, Faculty of Science Semlalia, Cadi Ayyad University, Marrakech 40000, Morocco; mohamed.aitelmokhtar@gmail.com (M.A.-E.-M.); a.meddich@uca.ma (A.M.); 3 Department of Biology, Faculty of Sciences and Techniques Mohammedia, Hassan II University, Casablanca, Mohammedia 20000, Morocco; 4 Laboratory of Materials, Processes, Environment, and Quality, National School of Applied Sciences of Safi, Cadi Ayyad University, Safi 46000, Morocco; abouliatim.younes@gmail.com; 5 Fertilizers Unit, OCP Group, Mohammed VI Polytechnic University, Jorf Lasfar 24025, Morocco; m.khouloud@ocpgroup.ma; 6 Center of Excellence in Soil and Fertilizer Research in Africa (CESFRA), AgroBioSciences, Mohammed VI Polytechnic University, Ben Guerir 43150, Morocco

**Keywords:** glass, phosphate, chemical durability, growth, yield, *Triticum durum*

## Abstract

Four different phosphate glass formulations (F_0_, F_1_, F_2_, and F_3_) were developed according o wheat nutrient requirements to be used as controlled-release fertilizers. These glasses contain macro-elements (P_2_O_5_-K_2_O-CaO-MgO), with the addition of microelements (Fe-Mn-Zn-B-Cu-Mo) in each formulation. The effects of these elements’ addition on thermal properties, glass structure, and dissolution behaviors were investigated. Results showed that these glasses are composed essentially of metaphosphate chains and that the addition of micronutrients could change the chemical durability of phosphate glasses. A greenhouse experiment was performed using wheat (*Triticum durum* L.) to evaluate the efficiency of the four glasses, with or without application of chemical nitrogen (N) (N + VF and VF, respectively). The different formulas were tested using two rates of 0.3 and 1 g per plant. In addition to the vitreous fertilizer formulations, two other treatments were applied: control treatment with no amendment and Nitrogen-Phosphorus-Potassium treatment with the application of the conventional fertilizers on the base of optimal rates. After four months of cultivation, vitreous fertilizers application significantly improved growth (7% to 88%), photosynthetic (8% to 49%) parameters, and yield (29% to 33%) compared to NPK treatment and to the control. It has been found that formulas F_1_, F_2_, and F_3_ may constitute a potential alternative to conventional fertilization due to their positive impact on wheat production and can be used in practice as an environmentally controlled-release fertilizer.

## 1. Introduction

The world population continues to increase, and at the existing rate of growth, it is expected to increase by over a third, or 2.3 billion people, between 2009 and 2050 [1]. This rate of growth is much slower than the one noticed in the past four decades, during which it grew by around 90% or 3.3 billion people [2]. Almost all of this growth is predicted to occur in developing countries [3]. These statistics mean that it is necessary to raise overall food production by around 70% to nourish a world population of 9.1 billion people in 2050 [4]. The market demand for food would require almost double in developing countries [5]. As a result, the production of several key basic commodities has significantly increased. To ensure nutritional security, cereals’ annual production, especially wheat, should increase by nearly one billion tons [6].

Wheat is considered a multipurpose crop due to its important utilization as human and animal food [7]. In this regard, wheat production is currently considered a great challenge for countries worldwide to maintain food security [8]. In Morocco and the other countries located in North Africa, wheat productivity is affected by various abiotic and biotic constraints, such as drought, high temperatures, leaf rust, severe imbalances in soil fertility (absence of essential nutrients and/or micro-organisms), and unfavorable soil physical characteristics especially degradation of agricultural soil resources which is already seriously limiting the production of crops in these countries [9,10,11]. Morocco’s wheat production in 2018 has been estimated to be 7,320,620 t with an area of cultivation of 2,842,748 ha [12]. To meet its needs for the same year, Morocco has imported 3,946,570 t of wheat to become the world’s 14th largest importer of this cereal [12].

For this, agriculture faces multiple challenges in the 21st century: to nourish a growing population with less arable lands, it has to produce more food force, and also it has to participate in the overall development in the many agriculture-dependent developing countries [13]. Furthermore, agriculture must adopt more efficient and eco-friendly production techniques because the use of chemical products, such as herbicides, insecticides, and fungicides, to increase agricultural production has become more harmful to plants’ health and the physicochemical quality of soils [14]. Moreover, the overuse of conventional fertilizers implies a large amount of nutrients in soils, leading to a high release velocity in such a way that plants cannot absorb and consume them [15]. The unconsumed released nutrients may also be adsorbed and retained either on the outer surface or within the pores of soil particles. Most of the nutrients pass to rivers or lakes, contaminating drinking water, and causing eutrophication [16].

Vitreous controlled-release fertilizers are considered one of the most promising solutions to increase crop yields without any environmental problems [17]. These fertilizers ensure the presence and availability of nutrient elements over time [15]. Consequently, the soil nutrients will be in adequate quantities but contained within exact and controllable limits, depending on crops requirement and development stage [15]. Generally, these nutrient elements are classified into three categories: primary elements, i.e., Nitrogen (N), Phosphorus (P) and Potassium (K); secondary elements, i.e., Calcium (Ca) and Magnesium (Mg); and microelements, i.e., Boron (B), Zinc (Zn), Manganese (Mn), Iron (Fe), Copper (Cu) and Molybdenum (Mo). This classification depends on the amount of elements absorbed by the crops, not on their function since they are all indispensable for the plants’ balanced growth. Glasses (especially phosphate glasses) have the ability to incorporate the majority of these nutrients, making it possible to develop fertilizers that provide plants with all that is needed to produce crops with high nutritional value [18].

For vitreous fertilizers, the controlled release rate of nutrients, which is the most distinguished property with conventional fertilizers, is principally linked to the chemical composition of glass [19]. It can be adjusted to have fertilizers that can dissolve quickly or maintain their activity for a long period, depending on plants’ requirements. Several microelements, such as Mn, Fe, Zn, Mo, and CuO, have been proposed to improve phosphate glasses’ chemical resistance [20,21,22,23,24].

In this study, four phosphate glass formulations (F_0_-F_1_-F_2_-F_3_) were established according to wheat nutrient requirements: F_0_ contains only major nutrients (P_2_O_5_-K_2_O-CaO-MgO), iron was added in F_1_ and manganese in F_2_, while F_3_ incorporates all microelements (Fe-Mn-Zn-B-Cu-Mo) necessary for wheat growth. This work focused on the effect of these elements on glass thermal properties, structure, and dissolution behaviors in order to assess their appropriateness of being applied as controlled-release fertilizers. In addition, an agronomic valorization was carried out to assess the effectiveness of the elaborated vitreous fertilizers on wheat growth, photosynthesis, and yield in comparison to non-amended and conventional fertilizers treatments under greenhouse conditions.

## 2. Materials and Methods

### 2.1. Glass Synthesis

**Figure 1:**
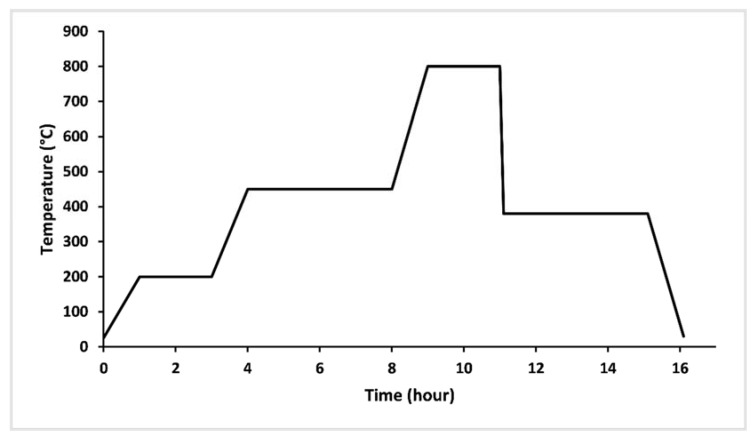
Thermal profile used to elaborate glasses.

The vitreous fertilizers were elaborated by the melt quench technique, using CaCO_3_, K_2_CO_3_, NH_4_H_2_PO_4_, MgO, Fe_2_O_3_, MnO, ZnO, H_3_BO_3_, CuO, and MoO_3_ as raw materials. The appropriate amounts of batch constituents were accurately weighed, drily milled to a fine powder and thoroughly mixed using an agate mortar, and then placed in an alumina crucible. The batches were thermally treated at 200 °C for 2 h and 450 °C for 4 h to eliminate CO_2_, H_2_O, and NH, and form the starting materials’ decomposition and prevent NH_4_H_2_PO_4_ foam. The melting stage lasted 2 h at 800 °C, as shown in Figure 1 [19].

The melted samples were taken out of the furnace and quenched in the air by pouring on a carbon mold. All the glasses were directly annealed at 10 °C below their transition temperature (T_g_) for about 4 h and then cooled slowly to ambient temperature. X-ray diffraction analysis was used to confirm the amorphous character of the glasses (PANAnalytical XPERT diffractometer working at 40 kV/200 mA, the angular range 10–70° (2θ) was scanned with a step size of 0.07° (2θ) and counting time of 5 s/step). The resulting glass compositions were examined using Inductively Coupled Plasma Optical Emission spectroscopy (ICP-OES Ultima Expert, Horiba Inc., Burlington, ON, Canada).

### 2.2. Thermal Analysis

Differential Thermal Analyzer was used to study the thermal properties of the vitreous fertilizers (DTA, Labsys Evo 1600, SETARAM). The process consists of heating ~30 mg of glass sample powder in a platinum crucible from ambient temperature to 800 °C at a heating rate of 10 °C min^−1^. The glass transition temperature (T_g_), the onset crystallization temperature (T_c,on_), peak crystallization temperatures (T_c_), the melting temperatures (T_f_) and the liquidus temperature (T_liq_) were recorded for all the samples.

Glass stability is described in terms of resistance to the crystallization of glass during heating and processes involving the reforming of existing glass. Hruby suggested that a parameter, K_H_, indicates glass stability against crystallization [25]. This parameter is defined by K_H_ = (T_c,on_ − T_g_)/(T_liq_ − T_c,on_). According to Hruby, glasses with higher values of K_H_ indicate higher stability against crystallization on heating and, apparently, higher vitrifiability on cooling.

### 2.3. Density Measurements

The glass’s density was measured at ambient temperature applying the Archimedes method using diethyl-ortho-phthalate as the buoyant liquid. The measurements were managed in accordance with the standard test method for the density of glass by buoyancy (ASTM C693). The samples mass was measured both in air and after immersion in diethyl-ortho-phthalate. The density was calculated from the following equation [26]:

ρ_glass_ = m_glass_/(m_glass_ + (m_ortho_ − m_(ortho + glass)_) × ρ_ortho_

with:

ρ = Density
m_glass_ = mass of glass measured in air
m_ortho_ = mass of diethyl-ortho-phthalate only
m_(ortho + glass)_ = mass of glass immersed in diethyl-ortho-phthalate
ρ_ortho_ = 1.11422 g/cm^3^

In order to obtain an average density value, the measurements were carried out three times.

The molar volume (V_M_) was determined from the density value and molecular glass weight of the batch composition using the equation: V_M_ = ρ_glass_/M_glass_ with M_glass_ as the glass’s molar mass [27].

### 2.4. Characterization of Glass Structure

Raman spectroscopy, and Fourier Transform infrared spectroscopy were used to study glass structure.

The Raman spectrum was obtained by analyzing a fine powder of glass using the Confotec MR520 Raman Confocal Microscope, with an Argon-ion laser emitting 514 nm as an excitation source. The spectra were obtained in the range 400–4000 cm^−1^ over an average of 128 scans and 1 s exposure time in the micro Raman compartment with a 10× objective.

FTIR spectra were obtained applying the KBr technique, using a spectrometer Bruker VERTEX 70, in the 400–4000 cm^−1^ domain, with a resolution of 4 cm^−1^, and 32 scans for each determination. Finely round glasses were mixed with pulverized KBr with a ratio (0.01/0.99 g), respectively. The weighted mixtures were subjected to a pressure of 6 t/cm^2^ to produce homogeneous discs. To avoid moisture attack, The FTIR spectra were measured immediately after preparing the mixture discs.

### 2.5. Glass Dissolution

Each glass sample’s chemical durability was defined from its dissolution rate (D_R_) in distilled water. The glass samples were pulverized and sieved to particle sizes between 1 and 2 mm. One gram of glass grains was placed in a vial containing 20 mL of distilled water with an initial pH of 6.5 [19].

To study the release rate of the glasses versus time, several samples were prepared and then suspended in a thermostatic bath maintained at temperature = 25 ± 1 °C for 1 to 35 days. The specimens were taken out at various time points, residual glass samples were filtered from leachate solutions, dried at 90 °C for 10 h, and then weighted using an analytic balance sensitive (±0.1 mg) (Shimadzu AW220).

Their dissolution rates were calculated using the following formula [19]:

$$D_{R}=\frac{W_{i}-W_{t}}{W_{i}}\times 100,$$

where *W_i_* is the sample’s initial weight, and *W_t_* is the sample’s weight after *t* days.

pH and ion measurements were carried out at the same time as the weight loss measurement took place, using a pH meter (Adwa-AD8000), and ICP-OES, respectively.

### 2.6. Agronomic Valorization of Vitreous Fertilizers

#### 2.6.1. Plant Material and Experimental Design

A greenhouse experiment was performed to evaluate the effect of the prepared vitreous fertilizers on wheat growth in the greenhouse with a day/night cycle of 16/8 h, 25.5 °C temperature average, 68.5% relative humidity average, and 410 μm^−2^ s^−1^ photon flux density average. The experiment was performed using two rates of the prepared vitreous fertilizers (VF R1 = 0.3 and VF R2 = 1 g/plant) compared with traditional mineral fertilizer (NPK). NPK fertilizer was added based on the recommendations of the Ministry of Agriculture and Fisheries [28].

The soil sample used in this experiment was taken from Saada district (10 Km Southwest of Marrakesh, Morocco) and was characterized by a pH value of 7.92; electrical conductivity (EC), 1.72 mS cm^−1^; available phosphorus, 31 mg kg^−1^; organic matter, 1.3%; and total organic carbon, 0.80%. The texture of this soil was sandy clay loam. 

Seeds of *Triticum durum* L. cv. Carioca underwent a 10 min sterilization using a 10% sodium hypochlorite solution and were rinsed several times with sterile distilled water. The germination test was performed in plastic dishes containing a sterile filter paper disk with incubation for seven days at 28 °C in the dark. One-week wheat seedlings were later transplanted into plastic pots (8 cm × 8 cm × 25 cm) (1 seedling/pot) containing 1.9 kg of soil.

The recommended doses of chemical fertilizer (NPK) were 140 kg N/ha as ammonium nitrate + 80 kg P_2_O_5_/ha as superphosphate + 50 kg K_2_O/ha as potassium sulphate. 

The experiment was designed in 18 treatments crossing four vitreous fertilizer levels (F_0_, F_1_, F_2,_ and F_3_) with two rates (0.3 and 1 g/plant) and two nitrogen fertilizer applications (0 and 1.4 g N/pot) besides NPK and control treatments. Pots of the different treatments were randomly disposed with ten replicates for each treatment (180 pots in total). Watering was done with the same amount of distilled water twice a week.

#### 2.6.2. Growth Parameters

At harvest (four months from germination), the following measurements were recorded: ears, shoot and root dry and fresh weights (g/plant), plant height (cm), root length (cm), leaf area (cm^2^), number of leaves, the weight of 1000 grain, and weight and number of grains (g/plant). The plants’ fresh weights were determined directly after the harvest, while dry weights were measured after the samples were kept at 105 °C for 24 h.

#### 2.6.3. Photosynthetic Efficiency and Stomatal Conductance

Measurements of these two parameters were carried out on fully expanded leaves from the third rank from five plants per treatment. Four measurements were taken from different parts of each leaf and their average was considered as one replicate. 

Chlorophyll fluorescence traits were assessed using a portable fluorometer (Opti-sciences OSI 30p). Leaf clips were used to keep the leaves in the dark for 30 min and then the measurements were recorded. Chlorophyll fluorescence was assessed as F_v_/F_m_ ratio where F_v_ = F_m_ − F_0_ and F_0_ and F_m_ are initial and maximum fluorescence respectively [29]. Stomatal conductance (gs) measurements were taken on a sunny day before harvest using a porometer system (Leaf Porometer LP1989, Decagon Device, Inc., Washington, DC, USA).

### 2.7. Statistical Analysis

The presented data are mean values based on three to five replicates ± standard error (SE) per treatment. SPSS software (IBM Corp. Released 2013. IBM SPSS Statistics for Windows, Version 22.0. Armonk, NY, USA: IBM Corp.) package for Windows was used to perform statistical analysis. All data were subjected to one-way analysis of variance (ANOVA) and the differences among means were assessed using Duncan’s test calculated at *p* < 0.05.

## 3. Results and Discussion

### 3.1. Glass Formation

**Figure 2:**
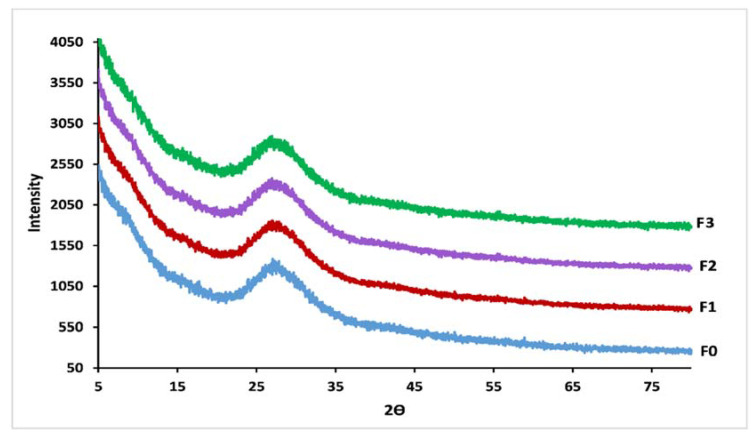
XRD patterns for F_0_, F_1_, F_2_ and F_3_ glasses.

As shown in Figure 2, no sharp peak was observed in the XRD patterns which confirms the amorphous nature of the vitreous fertilizers [30]. The entire glasses showed a regular and homogeneous surface.

Few bubbles were observed, and all the obtained glasses were transparent. The formula F_0_ was colorless, while F_1_, F_2,_ and F_3_ were brown, purple, and green, respectively. The brown color of phosphate glasses suggested the presence of Fe^2+^ and Fe^3+^ ions [31,32], while the purple color indicated the presence of Mn^2+^ and Mn^3+^ ions [32,33]. The green color of the formula F_3_ results from the presence of several elements like copper, iron, manganese, and molybdenum.

The analyzed compositions of the vitreous fertilizers are shown in Table 1. Differences between nominal and the analyzed compositions were minor for all samples and are attributed to measurement errors and melting volatilization.

### 3.2. Thermal Behaviour

**Figure 3:**
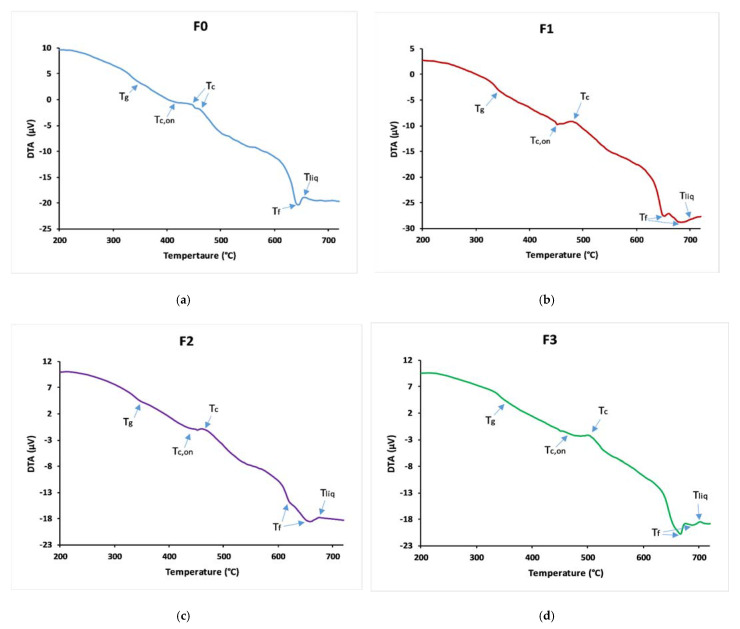
Differential scanning calorimetry curves of (**a**) F_0_, (**b**) F_1_, (**c**) F_2_, and (**d**) F_3_.

Figure 3 shows the thermal curves obtained from the differential thermal analysis, and Table 2 gives a summary of T_g_, T_c,on_, T_liq_, and K_H_ values of the samples. The three formulas containing microelements had higher glass transition, crystallization, and liquidus temperatures than F_0_. Glass F_3_ had higher values of T_g_ and T_liq_. For the two glasses F_1_ and F_2_, it can be seen that they had close values of T_g_, which is due to the Ionic Field Strength (IFS) of Fe and Mn (IFS = z/r^2^, where r is the ionic radius, and z is the valence cation), being IFS equal to 0.16 and 0.15 for Fe and Mn, respectively, according to Dietzel [34]. The increase in the glass transition temperature, which depends on the number and strength of the cross-links between oxygen atoms and the cation, and the density of covalent cross-linking, plays an important role in understanding the physical properties of glasses. This increase in Tg reflects a strengthening of the structure and increased network stability [35].

The DTA curves show multiple or broad crystallization and melting peaks. There is some evidence for the presence of multiple phases inside the glass matrix, or the network is constituted from different phosphate species [36]. The introduction of microelements in the phosphate glass matrix increased K_H_ from 0.1395 for F_0_ to 0.4391, 0.4839, and 0.5279 for F_1_, F_2,_ and F_3_ glasses, respectively, which reveals that the thermal stability of these glasses is greater than that of microelements-free glass samples, because the addition of these oxides creates cross-links between phosphate chains which reinforces the network [35].

### 3.3. Glass Density

Table 3 summarizes the measured densities of the studied glasses. The densities changed from 3.341 for F_0_ to 3.426 for F_3_, whereas the molar volumes varied from 33.18 to 32.66 cm^3^ mol^−1^. Density is sensitive to spatial arrangement and the nature of atoms [36,37]. Variations in glass density could illustrate the degree of structural compactness of the glass network. However, in this work, these changes were small and not likely to be significant because most of the microelements incorporated are glass modifiers (expect B_2_O_3_), principally placed in the holes in the vitreous network [38].

**Figure 4:**
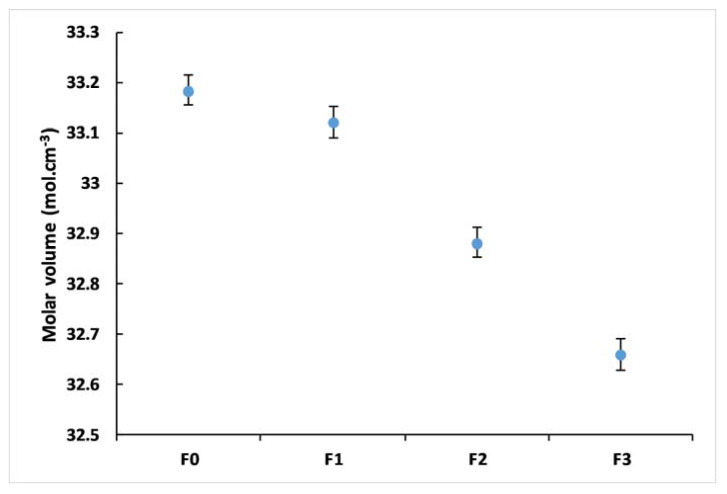
Molar Volume of F_0_, F_1_, F_2_, and F_3_ glasses.

The calculated molar volumes are shown in Figure 4. Molar volume, which compares volumes occupied by one mole of glass, is more sensitive to glass structure changes than density as it normalizes for atomic masses of glass components [27].

The decrease in molar volume by incorporating microelements reflects that the glass structure becomes more compact [35]. Furthermore, the increase in glass transition temperature (T_g_) accompanied by a decrease in the molar volume may reflect an overall increase in the glass network cross-linking [39].

### 3.4. Glass Structure

**Figure 5:**
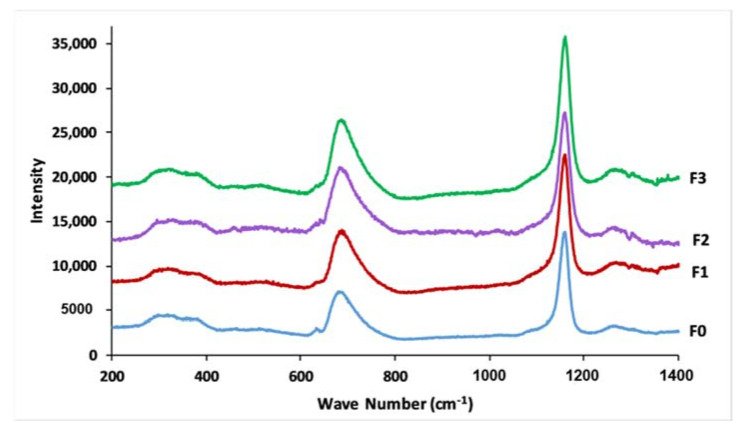
Raman spectra of prepared glasses.

The Raman spectra of the four phosphate glasses, in the range between 200 and 1400 cm^−1^, are presented in Figure 5. It is common knowledge that the phosphate network is built around PO_4_ tetrahedral units, which are classified depending on the number of bridging oxygens, using the Q^n^ designation, where “n” signifies the number of bridging oxygen atoms per tetrahedral unit (n = 0, 1, 2, 3) [40]. All Raman spectra are characterized by the existence of strong bands at around 1170 and 690 cm^−1^. Further weaker bands can be distinguished around 1270, 1100, 760, and 290–390 cm^−1^. The strong and broad band at 1170 cm^−1^ is assigned to the symmetric stretching mode of the PO_2_^−^ non-bridging bond in Q^2^ groups. The feature at 1270 cm^−1^ is related to the asymmetric stretch mode of PO_2_^−^, V_as_(PO_2_^−^) in Q^2^ groups. Q^1^ units appeared through two weak shoulders at 1100 cm^−1^ and 760 cm^−1^, which are attributed to the symmetric stretching vibration of terminal PO_3_^2−^ units, and to the symmetric stretching vibration of P–O–P, respectively. The band at 690 cm^−1^ is attributed to the symmetric stretching mode of the P–O–P in Q^2^ groups. Bands between 290 and 390 cm^−1^ could be related, respectively, to bending vibrations of PO_2_^−^ and PO_3_^2−^ [38,39,41,42,43].

**Figure 6:**
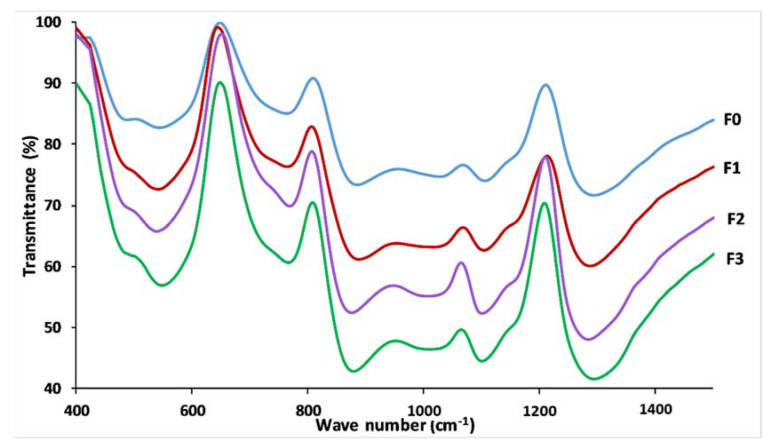
FTIR spectra of prepared glasses.

Figure 6 represents the FTIR spectra for the studied glasses in the range between 400 and 1400 cm^−1^, which shows no significant difference between the four formulas; this indicated that the prepared glasses have similar chemical functional groups and similar chemical bonding. The feature at around 1290 cm^−1^ is assigned to the asymmetric stretching of (PO_2_^−^) in the phosphate tetrahedron Q^2^, υ_as_ (PO_2_^−^). The FTIR bands observed at 1155–1160 cm^−1^ are characteristic of the symmetric stretching of (PO_2_^−^) in Q^2^ groups. The vibration of the band about 1100 cm^−1^ is attributed to the υ_s_ PO_3_^2−^ stretching vibrations, while the feature at 955–1080 cm^−1^ is attributed to the stretching vibration υ_as_ O–P–O band in the phosphate tetrahedron Q^1^. The two absorption peaks at 880 and 715 cm^−1^ are attributed to asymmetric and symmetric stretching of the P–O–P in Q^2^ groups, respectively. While the band at around 765 cm^−1^ is assigned to the P–O–P stretching vibrations Q^1^ species, bands between 550 and 480 cm^−1^ are assigned to bending vibration of O–P–O and PO_3_^2−^ bonds, respectively [26,38,44].

Raman and FTIR spectra suggest that the structure of these vitreous fertilizers resembles metaphosphates, and the network is composed essentially of Q^2^ units. However, the spectra also show the existence of Q^1^ units, generally result in the presence of shorter phosphate chains, which can explain the appearance of several T_c_ and T_f_ during thermal analyzes. Table 4 summarizes frequency ranges and assignments of the Raman and FTIR bands of the four glasses.

### 3.5. Dissolution Behavior

**Figure 7:**
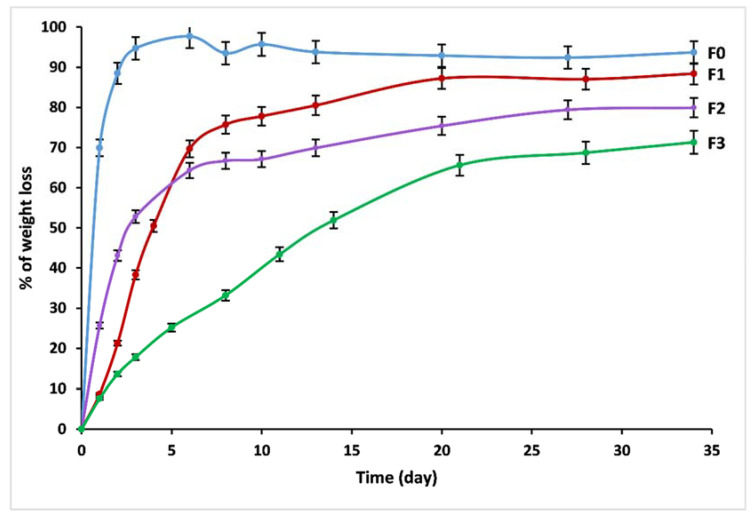
Trend of weight loss of F_0_, F_1_, F_2_ and F_3_ glasses.

With increasing dissolution time in distilled water, the vitreous fertilizers exhibit an increased D_R_, as revealed in Figure 7. Chemical bonds between glass modifiers and glass formers are created due to the vitrification process. Consequently, if the glass stays undissolved, those modifiers cannot be liberated.

The dissolution of phosphate glass is the result of a set of complex mechanisms that depends not only on its physicochemical properties but also on the leaching conditions [45]. When glass particles are in contact with water, processes of inter-diffusion, ion-exchange, reaction–diffusion, and hydrolysis take place. These processes involve three dissolution rate regimes: (i) Initial diffusion, which reflects the exchange between protons in leachate solution and glass network-modifier cations. At the beginning of dissolution, water particles permeate into the glass, mobile alkali modifier ions undergo diffusional ion exchange with protons in the solution; (ii) Hydrolysis process which involves the hydrolysis of P-O-M bonds (with M = P, Mg, Ca, Zn, Fe, etc.), constituting the network structure of a glass [46]. Hydrolysis changes the phosphate network by attacking bridging bonds in the interphase formed by mobile elements’ release; and (iii) Rate drop, which is a transition between the initial rate and residual dissolution rate, as a result of the gradual saturation of the solution. This saturation induces a gradual rate decrease until a residual dissolution rate where the glass dissolution rate attains a relatively constant value, and thermodynamic equilibrium is approached—i.e., the chemical affinity for dissolution decreases [45,46].

The initial dissolution rates V_0_ (V_0_ = $\frac{dm}{dt}$ of the linear part of the dissolution curves) are given in Table 5. The chemical resistance of the glass is mainly dependent on its chemical composition. Formula F_0_ showed the highest dissolution rate, while F_3_ showed the lowest dissolution rate, followed by F_2_ and F_1_. The initial diffusion and hydrolysis process for F_0_ lasted only two days, with an initial dissolution rate V_0_ = 0.69 g/day. Almost the entirety was dissolved in water within less than four days. The degradation rate was found to decrease for F_1_ and F_2_ by incorporating iron and manganese into the glass matrix. The initial dissolution rates for these glasses were 0.14 and 0.17 g/day, respectively. The initial diffusion and hydrolysis process lasted between four and six days.

Hasan et al. [47] have studied the chemical durability of P_2_O_5_-Fe_2_O_3_-Na_2_O-CaO-MgO glasses and reported that Fe_2_O_3_ addition leads to the creation of more hydration resistant Fe-O-P bonds instead of P-O-P bonds, which increased cross-linking between the phosphate chains and improved the chemical durability of the glass.

Ahmina et al. [48] suggested that by adding MnO to phosphate glasses, the chemical resistance was enhanced due to the increase in the cross-link between the phosphate chains by the formation of P–O–Mn bonds. These changes can be explained by the effect of cation substitution on the glass network structure. The addition of MnO causes the phosphate network to shrink and produce more entangled and networked metaphosphate chains.

In all the investigations above, MnO and Fe_2_O_3_ can both improve the durability of phosphate glasses; however, this study showed that Fe_2_O_3_ was much more effective in decreasing the initial degradation rate, while MnO had a greater effect on decreasing the residual rate. The admixture of Fe and Mn, in addition to other elements such as Zn, B, Cu, and Mo, in a phosphate glass network (F_3_) induces a rapid improvement in the chemical durability, which may be related to the strengthening of the bonds between non-bonding oxygen atoms and cations, leading to an overall network reticulation effect. The hydration process based on ion exchange between the cations in phosphate chains and water becomes thermodynamically less favorable with the increase of the cross-linking between the chains. Glass F_3_ has a V_0_ = 0.03 g/day; after 34 days, it had not yet reached the saturation stage, with a weight loss of only 71%.

**Figure 8:**
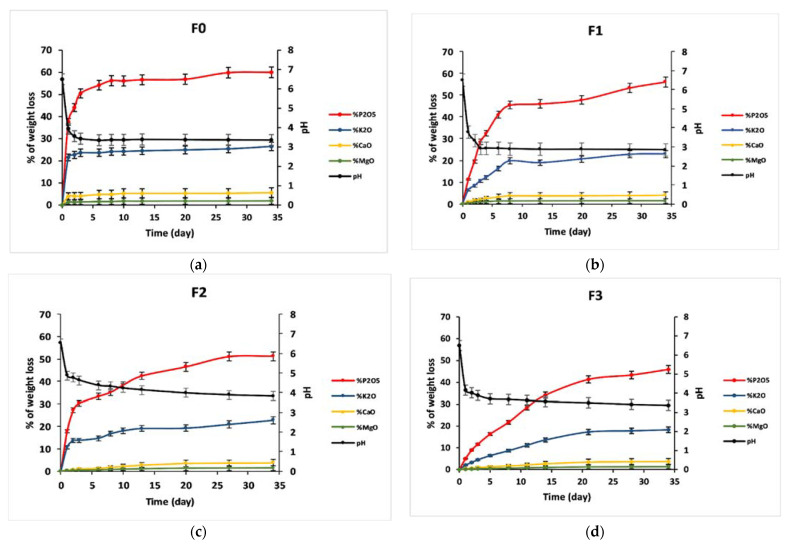
Percentage of glass constituents (macro-elements) analyzed in the leachate solutions (elements in the form of oxides) normalized to the initial glass weight and pH measurements versus time for (**a**) F_0_, (**b**) F_1_, (**c**) F_2,_ and (**d**) F_3_.

**Figure 9:**
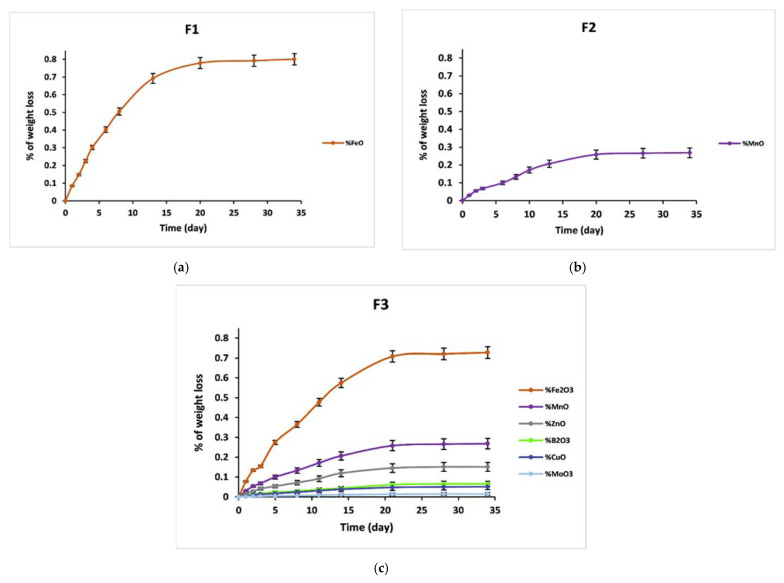
Percentage of glass constituents (micro-elements) analyzed in the leachate solutions (elements in the form of oxides) normalized to the initial glass weight for (**a**) F_1_, (**b**) F_2_, and (**c**) F_3_.

Amounts of released elements from vitreous fertilizers to the leachate solution were determined using the ICP-OES, in the form of oxides normalized to the initial glass weight, and the pH measurements are presented in Figure 8 and Figure 9. The percentage of released ions increased over time. For F_0_, amounts of P, K, Ca, and Mg in distilled water were significantly enhanced during the first two days of immersion. While for glasses F_1_, F_2,_ and F_3,_ the effect of the addition of microelements, which resulted in a slower release of ions in water, has been noted. For the four glass formulations, the presence of entire elements in the analyzed solutions, with a percentage comparable to the glass composition, suggests that the glasses dissolved congruently, and no selective leaching occurred [49]. The pH of the leachate solutions changed after immersion of glasses in distilled water. pH diminished linearly with dissolution time from 6.5 to attain the acidic range for all the studied fertilizers, then remained almost unchanged during periods of immersion. Previous studies showed the leachate solution’s pH varied with phosphate content of the immersed glass, with higher phosphorus contents in the solution resulting in lower pH values [50]. However, even though formula F_0_ releases more phosphorus, formula F_1_ achieves a lower pH value. This can be explained by the fact that with the addition of iron, the metaphosphate chains are broken into smaller groups of short-chain phosphates such as P_4_O_13_^6−^, P_3_O_10_^5−^ and P_2_O_7_^4−^, which are linked to iron through P–O–Fe bonds [51]. This phenomenon was not noted during the structural characterization by FTIR and Raman, which means that these short chains are in small quantities but have a remarkable effect on the pH.

### 3.6. Growth Parameters

The application of F_1_ treatments mainly improved plant height, fresh and dry shoot weight, fresh ear weight, and the number of grains per plant, and the F_2_ treatments mainly improved leaf area, fresh and dry root weight, and 1000 grain weight compared to the control and NPK treatments (Table 6). On the other hand, the F_0_ and F_3_ treatments increased the root length and dry ear weight_,_ respectively, compared to the control and NPK treatments. Ouis et al. [52] reported an improvement of ears, straw, grains, and maize yield under field conditions after applying vitreous fertilizers (SiO_2_, P_2_O_5_, K_2_O, Fe_2_O_3_, CuO). In addition, Abou-Baker et al. [53] reported the same results using vitreous fertilizers containing the same elements in addition to ZnO and CuO.

Considering the maximum values of improvement, fresh and dry shoot weight, fresh ear weight, and the number of grains per plant showed a maximum improvement with the application of F_1_ (F_1_ R1 (30% to 58%) and F_1_ R2 (18% to 61%)) (Table 6). On the other hand, plant height and root length, fresh root weight, and weight of 1000 gain showed a maximum increase after the application of F_2_ (F_2_ R1 (23% to 64%) and F_2_ R2 (23% to 159%)). In addition, root and ear dry weights and grain weight per plant showed a maximum improvement after the application of F_3_ (F_3_ R1 + N (63 to 188%) and F_3_ R2 (85 to 140%)). The leaf area exhibited a maximum improvement after the application of F_0_ R2 (28%). The positive effect of the vitreous fertilizers on growth traits (especially F_1_ and F_2_) could be explained by the high rates of release of different mineral elements contained in the vitreous fertilizers [54].

### 3.7. Photosynthetic Parameters

The stomatal conductance (gs) and photosystem II efficiency (F_v_/F_m_) were increased by 32% and 13%, respectively, with the application of NPK fertilizer compared to the control. The gs was increased by 70% in plants treated with vitreous fertilizers (34% for F_0_, 45% for F_1_, 47% for F_2,_ and 107% for F_3_) (Table 6), while F_v_/F_m_ was increased by 14% with the application of these fertilizers (11% for F_0_, 13% for F_1_, 16% for F_2,_ and 18% for F_3_). The F_2_ provided the highest percentages of improvement of these two parameters (151% (F_2_ R1) and 116% (F_2_ R2 + N) for gs and 24% (F_2_ R2) for F_v_/F_m_). The improvements in these photosynthetic attributes by the application of vitreous fertilizers could be explained by the key role of these amendments in providing essential elements such as potassium, magnesium, copper, iron, and manganese, which are involved in many photosynthetic related processes and biomolecules, including stomata movements and photosynthetic pigments and enzymes. Ion et al. [55] demonstrate that the application of vitreous fertilizers improved grapevine nutrition, in particular K and Mg uptake, which can stimulate many metabolism pathways, such as the regulation of stomatal exchanges as well as the balance of hormones such as ABA and thereby the photosynthesis functioning [56]. The absorption of the essential nutrients included in the vitreous fertilizers boosts wheat growth and yield performances.

Based on the number of the improved parameters and the maximum values of this improvement, F_1_, F_2_, and F_3_ were distinguished in comparison to the control, NPK and F_0_ treatments especially with R2 application (1 g/plant). It seems that these three effective formulations could be suitable for a large-scale application in the open field to further investigate the performance of the applied vitreous fertilizers.

## 4. Conclusions

Physico-chemical properties, structure, and dissolution behaviors of four phosphate glasses, elaborated according to wheat nutrient requirements, have been investigated in this study. It was confirmed that the prepared vitreous fertilizers are amorphous, Raman and FTIR spectra showed that their structure approaches metaphosphates, and the network is formed essentially of Q^2^ units. This study showed an increase in glass transition temperature (T_g_), the onset crystallization temperature (T_c_), liquidus temperature (T_f_), and glass stability accompanied by a decrease in the molar volume (V_M_) and glass dissolution with incorporating trace elements such as Fe_2_O_3_ and MnO. It was suggested that these behaviors are due to stronger cross-linking of the phosphate chains and the replacement of the easily hydrated P-O-P bond by a more chemically resistant M-O-P bond (M = Fe, Mn, Zn, Mo, etc.). Moreover, for all glasses, no selective ion leaching was observed, and the dissolution was congruent. The prepared vitreous fertilizers, in particular F_1_, F_2,_ and F_3_, showed a boosting effect on wheat growth, photosynthetic, and yield traits compared to non-amended and NPK treatments, suggesting the importance of considering the use of these fertilizers in large-scale application to improve crop production with no harm to the environment.

## Figures and Tables

**Table 1:** Nominal and analyzed compositions of the vitreous fertilizers.

Glass	Nominal Compositions									
	% P_2_O_5_	% K_2_O	% CaO	% MgO	% Fe_2_O_3_	% MnO	% ZnO	% B_2_O_3_	% CuO	% MoO_3_
F_0_	50.00	33.33	11.11	5.56	0.00	0.00	0.00	0.00	0.00	0.00
F_1_	50.81	32.26	10.75	5.38	0.81	0.00	0.00	0.00	0.00	0.00
F_2_	50.00	32.89	10.96	5.48	0.00	0.66	0.00	0.00	0.00	0.00
F_3_	50.72	31.51	10.50	5.25	0.79	0.63	0.32	0.16	0.11	0.02
	**Analyzed Compositions**									
	**% P_2_O_5_**	**% K_2_O**	**% CaO**	**% MgO**	**% Fe_2_O_3_**	**% MnO**	**% ZnO**	**% B_2_O_3_**	**% CuO**	**% MoO_3_**
F_0_	50.17 ± 1.01	33.26 ± 0.83	11.07 ± 0.24	5.50 ± 0.17	0.00	0.00	0.00	0.00	0.00	0.00
F_1_	50.64 ± 1.00	32.43 ± 0.90	10.52 ± 0.33	5.57 ± 0.17	0.84 ± 0.02	0.00	0.00	0.00	0.00	0.00
F_2_	49.94 ± 1.22	33.07 ± 0.82	10.78 ± 0.23	5.50 ± 0.21	0.00	0.71 ± 0.01	0.00	0.00	0.00	0.00
F_3_	50.99 ± 1.19	31.45 ± 0.78	10.37 ± 0.28	5.23 ± 0.15	0.77 ± 0.02	0.64 ± 0.01	0.26 ± 0.01	0.15 ± 0.02	0.13 ± 0.02	0.01 ± 0.01

**Table 2:** Glass transition (T_g_) crystallization (T_c,on_) melting (T_liq_) temperatures and K_H_ of prepared glasses.

Glass	F_0_	F_1_	F_2_	F_3_
T_g_ (°C)	340 ± 2	345 ± 2	348 ± 1	353 ± 1
T_c,on_ (°C)	417 ± 5	453 ± 2	453 ± 3	573 ± 2
T_liq_ (°C)	658 ± 3	699 ± 4	670 ± 2	709 ± 3
K_H_	0.319 ± 0.002	0.4391 ± 0.001	0.484 ± 0.002	0.528 ± 0.002

**Table 3:** Density of F_0_, F_1_, F_2_, and F_3_ glasses.

Glass	F_0_	F_1_	F_2_	F_3_
Density	3.341 ± 0.002	3.382 ± 0.003	3.371 ± 0.003	3.426 ± 0.005

**Table 4:** Assignments and frequency ranges (cm^−1^) of the FTIR and Raman bands of the prepared glasses.

Wave Number (cm^−1^)								Assignment
F_0_		F_1_		F_2_		F_3_		-
FTIR	Raman	FTIR	Raman	FTIR	Raman	FTIR	Raman	
1296	1270	1288	1269	1286	1272	1296	1269	Vas (PO_2_^−^), Q^2^
1159	1174	1155	1171	1151	1174	1159	1172	Vs (PO_2_^−^), Q^2^
1107	1103	1109	1101	1105	1103	1105	1101	Vs (PO_3_^2−^), Q^1^
962–1074	-	956–1074	-	958–1072	-	956–1072	-	Vas (PO_2_^−^), Q^1^
885	-	889	-	877	-	879	-	Vas (P-O-P), Q^2^
761	758	767	758	767	759	771	760	Vs (P-O-P), Q^1^
717	691	715	691	719	690	719	689	Vs (P-O-P), Q^2^
557	382	547	385	543	383	549	386	δ(PO_2_^−^)
487	296–332	491	294–336	486	301–340	491	290–335	δ(PO_3_^2−^)

* Abbreviations: as, asymmetric; s, symmetric; V, stretching; δ, bending.

**Table 5:** Initial dissolution rate of F_0_, F_1_, F_2_, and F_3_ glasses.

Glass	F_0_	F_1_	F_2_	F_3_
V_0_ (g/day)	0.69	0.14	0.17	0.03

**Table 6:** Effects of vitreous fertilizers on the growth, physiology, and yield of wheat after four months of culture.

Fertilizer Treatment	Plant Height (cm)	Root Length (cm)	Number of Leaves	Leaf Area (cm^2^)	Shoot Fresh Weight (g)	Shout Dry Weight (g)	Root Fresh Weight (g)	Root Dry Weight (g)	Ear Fresh Weight (g)	Ear Dry Weight (g)	Total Grain Weight/Plant (g)	Number of Grain/Plant	1000 Grain Weight (g)	Stomatal Conductance (mmol m^−2^ s^−1^)	F_v_/F_m_
**Control**	60.43 ± 1.34 ^j^	17.33 ± 0.58 ^i^	5.33 ± 0.58 ^d^	17.85 ± 0.26 ^h^	2.27 ± 0.61 ^f^	0.5 ± 0.13 ^g^	0.95 ± 0.20 ^h^	0.23 ± 0.05 ^h^	1.18 ± 0.27 ^f^	0.68 ± 0.12 ^h^	0.46 ± 0.05 ^ef^	18.67 ± 3.21 ^f^	16.78 ± 0.87 ^i^	17.63 ± 1.66 ^j^	0.65 ± 0.03 ^g^
**NPK fertilizer**	67.27 ± 0.25 ^i^	18.67 ± 0.58 ^ghi^	7.00 ± 0.00 ^a^	28.60 ± 2.20 ^d–g^	3.44 ± 0.32 ^e^	0.88 ± 0.05 ^ef^	1.83 ± 0.10 ^de^	0.26 ± 0.11 ^gh^	1.99 ± 0.28 ^e^	0.74 ± 0.15 ^gh^	0.44 ± 0.10 ^f^	25.67 ± 4.04 ^b–f^	27.79 ± 4.22 ^cde^	23.37 ± 2.69 ^i^	0.74 ± 0.03 ^a–f^
**F_0_ R1**	75.17 ± 2.75 ^d–g^	26.33 ± 1.15 ^a^	6.00 ± 0.00 ^bcd^	25.39 ± 2.94 ^fg^	3.75 ± 0.21 ^de^	1.20 ± 0.08 ^bc^	1.65 ± 0.15 ^ef^	0.43 ± 0.02 ^c–f^	1.99 ± 0.30 ^e^	0.87 ± 0.15 ^e–h^	0.47 ± 0.03 ^def^	25.67 ± 2.08 ^b–f^	22.18 ± 3.18 ^e–i^	22.33 ± 0.38 ^i^	0.68 ± 0.05 ^efg^
**F_0_ R1 + N**	82.13 ± 2.73 ^d–g^	23.00 ± 1.00 ^b–e^	6.33 ± 0.58 ^abc^	26.90 ± 2.71 ^efg^	4.52 ± 0.21 ^a–d^	1.23 ± 0.10 ^bc^	1.51 ± 0.18 ^efg^	0.33 ± 0.06 ^fgh^	2.42 ± 0.09 ^b–e^	1.06 ± 0.08 ^c–f^	0.71 ± 0.04 ^bcd^	28.33 ± 3.79 ^a–d^	24.02 ± 3.27 ^d–h^	25.33 ± 3.12 ^ghi^	0.80 ± 0.01 ^ab^
**F_0_ R2**	82.10 ± 1.13 ^ab^	20.00 ± 0.00 ^f–i^	6.00 ± 0.00 ^abc^	39.42 ± 2.48 ^a^	4.49 ± 0.28 ^a–d^	1.37 ± 0.09 ^ab^	1.55 ± 0.35 ^efg^	0.49 ± 0.07 ^bcd^	2.88 ± 0.11 ^abc^	1.12 ± 0.13 ^b–e^	0.78 ± 0.15 ^abc^	30.67 ± 1.15 ^abc^	26.08 ± 2.94 ^c–g^	24.60 ± 1.22 ^ghi^	0.72 ± 0.05 ^efg^
**F_0_ R2 + N**	74.00 ± 2.21 ^d–h^	24.67 ± 1.53 ^abc^	6.33 ± 0.58 ^abc^	27.15 ± 1.93 ^efg^	5.01 ± 0.80 ^ab^	1.34 ± 0.01 ^ab^	1.58 ± 0.11 ^efg^	0.34 ± 0.07 ^e–h^	2.39 ± 0.19 ^b–e^	0.82 ± 0.11 ^fgh^	0.49 ± 0.13 ^def^	24.33 ± 5.13 ^c–f^	20.10 ± 1.83 ^ghi^	22.60 ± 2.21 ^i^	0.71 ± 0.03 ^d–g^
**F_1_ R1**	80.23 ± 4.20 ^abc^	24.67 ± 2.89 ^abc^	5.67 ± 0.58 ^cd^	34.89 ± 2.52 ^a–d^	5.10 ± 0.85 ^a^	1.33 ± 0.19 ^ab^	1.87 ± 0.55 ^de^	0.32 ± 0.03 ^fgh^	3.15 ± 0.32 ^a^	1.33 ± 0.12 ^bc^	0.88 ± 0.12 ^abc^	33.33 ± 2.52 ^a^	26.43 ± 1.66 ^c–f^	27.27 ± 0.59 ^efg^	0.78 ± 0.06 ^a–d^
**F_1_ R1 + N**	77.00 ± 2.78 ^c–f^	23.33 ± 0.76 ^bcd^	6.33 ± 0.58 ^abc^	32.79 ± 4.45 ^a–e^	4.94 ± 0.24 ^abc^	1.32 ± 0.05 ^ab^	1.54 ± 0.17 ^efg^	0.44 ± 0.10 ^c–f^	3.09 ± 0.38 ^a^	1.25 ± 0.21 ^bcd^	0.81 ± 0.09 ^abc^	30.33 ± 3.21 ^abc^	24.06 ± 2.44 ^d–h^	28.37 ± 1.27 ^ef^	0.71 ± 0.01 ^c–g^
**F_1_ R2**	82.77 ± 2.68 ^ab^	23.33 ± 0.58 ^bcd^	7.00 ± 0.00 ^a^	38.13 ± 3.95 ^ab^	5.26 ± 0.69 ^a^	1.43 ± 0.13 ^a^	2.29 ± 0.32 ^c^	0.54 ± 0.05 ^abc^	3.08 ± 0.34 ^abc^	1.34 ± 0.14 ^bc^	0.91 ± 0.15 ^ab^	30.33 ± 0.57 ^abc^	30.41 ± 4.75 ^c^	22.93 ± 1.56 ^i^	0.79 ± 0.04 ^abc^
**F_1_ R2 + N**	74.23 ± 1.07 ^d–h^	22.00 ± 2.65 ^c–f^	6.00 ± 0.00 ^bcd^	24.96 ± 1.28 ^fg^	4.91 ± 0.37 ^abc^	1.30 ± 0.07 ^ab^	1.55 ± 0.16 ^efg^	0.28 ± 0.01 ^gh^	2.93 ± 0.22 ^abc^	1.12 ± 0.15 ^b–e^	0.76 ± 0.10 ^abc^	30.00 ± 2.65 ^abc^	19.97 ± 0.53 ^hi^	23.83 ± 1.60 ^hi^	0.68 ± 0.03 ^fg^
**F_2_ R1**	82.87 ± 2.73 ^a^	25.00 ± 2.00 ^ab^	6.33 ± 0.58 ^abc^	34.11 ± 6.20 ^abc^	4.54 ± 0.57 ^a–d^	1.35 ± 0.12 ^ab^	2.80 ± 0.20 ^b^	0.61 ± 0.09 ^ab^	2.77 ± 0.11 ^abc^	1.23 ± 0.09 ^bcd^	0.86 ± 0.11 ^abc^	22.33 ± 5.69 ^def^	45.54 ± 4.08 ^a^	44.33 ± 0.59 ^a^	0.77 ± 0.06 ^a–e^
**F_2_ R1 + N**	69.77 ± 0.97 ^hi^	17.33 ± 0.58 ^i^	7.00 ± 0.00 ^a^	32.22 ± 5.57 ^b–f^	3.41 ± 0.69 ^e^	0.92 ± 0.04 ^ef^	1.23 ± 0.09 ^fgh^	0.31 ± 0.06 ^fgh^	2.15 ± 0.39 ^de^	0.96 ± 0.19 ^d–h^	0.64 ± 0.14 ^c–f^	27.67 ± 1.53 ^a–d^	20.78 ± 0.67 ^fgh^	28.70 ± 0.87 ^ef^	0.76 ± 0.02 ^a–f^
**F_2_ R2**	82.90 ± 4.37 ^a^	27.00 ± 2.00 ^a^	7.00 ± 0.00 ^a^	36.07 ± 2.19 ^abc^	4.75 ± 0.38 ^abc^	1.29 ± 0.12 ^ab^	4.75 ± 0.38 ^a^	0.51 ± 0.03 ^abc^	2.99 ± 0.27 ^ab^	1.17 ± 0.36 ^bcd^	0.80 ± 0.14 ^abc^	26.00 ± 3.60 ^a–e^	39.04 ± 8.53 ^b^	26.50 ± 1.70 ^fgh^	0.82 ± 0.04 ^a^
**F_2_ R2 + N**	78.47 ± 2.47 ^a–d^	21.33 ± 2.31 ^d–g^	7.00 ± 1.00 ^a^	31.69 ± 3.32 ^b–f^	4.63 ± 0.58 ^a–d^	1.25 ± 0.06 ^abc^	2.10 ± 0.09 ^cd^	0.46 ± 0.08 ^cde^	2.93 ± 0.36 ^abc^	1.31 ± 0.19 ^bc^	0.91 ± 0.14 ^ab^	32.67 ± 4.51 ^ab^	28.89 ± 1.23 ^cd^	38.03 ± 0.91 ^bc^	0.70 ± 0.07 ^efg^
**F_3_ R1**	76.87 ± 2.32 ^c–f^	18.20 ± 1.56 ^hi^	6.67 ± 0.58 ^ab^	25.45 ± 1.50 ^fg^	3.56 ± 0.16 ^e^	0.98 ± 0.12 ^def^	1.63 ± 0.11 ^ef^	0.38 ± 0.05 ^d–g^	2.35 ± 0.04 ^cde^	1.05 ± 0.04 ^c–f^	0.71 ± 0.02 ^bcd^	27.33 ± 2.08 ^a–e^	27.30 ± 1.76 ^cde^	37.17 ± 1.22 ^bcd^	0.74 ± 0.05 ^a–f^
**F_3_ R1 + N**	73.57 ± 2.06 ^e–h^	20.33 ± 0.57 ^e–h^	6.67 ± 0.57 ^ab^	30.30 ± 3.54 ^c–f^	3.33 ± 0.13 ^e^	0.96 ± 0.08 ^def^	1.14 ± 0.16 ^gh^	0.25 ± 0.04 ^gh^	2.63 ± 0.21 ^a–d^	2.13 ± 0.15 ^a^	0.71 ± 0.14 ^bcd^	31.00 ± 5.20 ^abc^	23.80 ± 1.97 ^d–h^	35.33 ± 0.78 ^cd^	0.78 ± 0.03 ^a–d^
**F_3_ R2**	78.03 ± 3.10 ^b–e^	20.67 ± 1.53 ^d–h^	7.00 ± 0.00 ^a^	35.60 ± 1.05 ^a–d^	4.15 ± 0.07 ^b–e^	1.10 ± 0.04 ^cd^	2.20 ± 0.19 ^cd^	0.62 ± 0.07 ^a^	2.96 ± 0.22 ^abc^	1.37 ± 0.04 ^b^	0.98 ± 0.05 ^a^	32.67 ± 1.15 ^ab^	30.14 ± 2.13 ^c^	39.00 ± 1.47 ^b^	0.81 ± 0.02 ^a^
**F_3_ R2 + N**	72.87 ± 3.07 ^fgh^	18.00 ± 1.00 ^hi^	7.00 ± 0.00 ^a^	22.84 ± 0.0.28 ^gh^	3.45 ± 0.37 ^e^	0.85 ± 0.05 ^f^	1.17 ± 0.05 ^gh^	0.37 ± 0.09 ^d–g^	2.43 ± 0.13 ^b–e^	0.99 ± 0.06 ^d–g^	0.70 ± 0.06 ^b–e^	28.00 ± 1.73 ^a–d^	24.86 ± 0.69 ^c–h^	34.50 ± 2.11 ^d^	0.76 ± 0.04 ^a–f^

* Control: treatment with no fertilizer addition, NPK fertilizer: treatment with traditional chemical fertilizers, F_0_ R1: treatment with formula 0 of vitreous fertilizers at 0.3 g/pot, F_0_ R1 + N: treatment with formula 0 of vitreous fertilizers at 0.3 g/pot combined with traditional chemical fertilizers, F_0_ R2: treatment with formula 0 of vitreous fertilizers at 1 g/pot, F_0_ R2 + N: treatment with formula 0 of vitreous fertilizers at 1 g/pot combined with traditional chemical fertilizers, F_1_ R1: treatment with formula 1 of vitreous fertilizers at 0.3 g/pot, F_1_ R1 + N: treatment with formula 1 of vitreous fertilizers at 0.3 g/pot combined with traditional chemical fertilizers, F_1_ R2: treatment with formula 1 of vitreous fertilizers at 1 g/pot, F_1_ R2 + N: treatment with formula 1 of vitreous fertilizers at 1 g/pot combined with traditional chemical fertilizers, F_2_ R1: treatment with formula 2 of vitreous fertilizers at 0.3 g/pot, F_2_ R1 + N: treatment with formula 2 of vitreous fertilizers at 0.3 g/pot combined with traditional chemical fertilizers, F_2_ R2: treatment with formula 2 of vitreous fertilizers at 1 g/pot, F_2_ R2 + N: treatment with formula 2 of vitreous fertilizers at 1 g/pot combined with traditional chemical fertilizers, F_3_ R1: treatment with formula 3 of vitreous fertilizers at 0.3 g/pot, F_3_ R1 + N: treatment with formula 3 of vitreous fertilizers at 0.3 g/pot combined with traditional chemical fertilizers, F_3_ R2: treatment with formula 3 of vitreous fertilizers at 1 g/pot, F_3_ R2 + N: treatment with formula 3 of vitreous fertilizers at 1 g/pot combined with traditional chemical fertilizers. Mean values in each column followed by the same letter did not differ significantly at *p* < 0.05 by Duncan’s test.

## Data Availability

Data is contained within the article.
